# Establishment of a Yeast Two-Hybrid-Based High-Throughput Screening Model for Selection of SARS-CoV-2 Spike-ACE2 Interaction Inhibitors

**DOI:** 10.3390/ijms26020678

**Published:** 2025-01-15

**Authors:** Dongsheng Li, Baoqing You, Keyu Guo, Wenwen Zhou, Yan Li, Chenyin Wang, Xiaofang Chen, Zhen Wang, Jing Zhang, Shuyi Si

**Affiliations:** 1 State Key Laboratory of Bioactive Substances and Functions of Natural Medicines, Institute of Medicinal Biotechnology, Chinese Academy of Medical Sciences & Peking Union Medical College, Beijing 100050, China; lids105@163.com (D.L.); ybq0606@163.com (B.Y.); gky3448644487@163.com (K.G.); zwwaoe@163.com (W.Z.); liyan@imb.pumc.edu.cn (Y.L.); wangchenyin@imb.pumc.edu.cn (C.W.); chenxiaofang@imb.pumc.edu.cn (X.C.); wangzhen@imb.pumc.edu.cn (Z.W.); 2 Research Team of Molecular Medicine, The First Clinical Medical School of Shanxi Medical University, Taiyuan 030001, China

**Keywords:** yeast-two hybrid system, high-throughput screening, spike protein, angiotensin-converting enzyme 2, protein–protein interaction inhibitor

## Abstract

The recent coronavirus disease 2019 (COVID-19) pandemic, caused by severe acute respiratory syndrome coronavirus 2 (SARS-CoV-2), has exerted considerable impact on global health. To prepare for rapidly mutating viruses and for the forthcoming pandemic, effective therapies targeting the critical stages of the viral life cycle need to be developed. Viruses are dependent on the interaction between the receptor-binding domain (RBD) of the viral Spike (S) protein (S-RBD) and the angiotensin-converting enzyme 2 (ACE2) receptor to efficiently establish infection and the following replicate. Targeting this interaction provides a promising strategy to inhibit the entry process of the virus, which in turn has both preventive and therapeutic effects. In this study, we developed a robust and straightforward assay based on the Yeast-Two Hybrid system (Y2H) for identifying inhibitors targeting the S-RBD-ACE2 interaction of SARS-CoV-2. Through high-throughput screening, two compounds were identified as potential entry inhibitors. Among them, IMB-1C was superior in terms of pseudovirus entry inhibition and toxicity. It could bind to both ACE2 and S-RBD and induce conformational change in the S-RBD+ACE2 complex. This is the first study to verify the feasibility of utilizing the Y2H system to discover potent SARS-CoV-2 inhibitors targeting the receptor recognition stage. This approach may also be applied in the discovery of other virus receptor recognition inhibitors.

## 1. Introduction

Since the beginning of the 21st century, three coronaviruses (CoVs) have crossed the species barrier to cause fatal pneumonia in humans, including the severe acute respiratory syndrome coronavirus (SARS-CoV) [1], the Middle-East respiratory syndrome coronavirus (MERS-CoV) [2], and SARS-CoV-2 [3], the causative agent of coronavirus disease 2019 (COVID-19). Although multiple vaccines have shown promising efficacy against the development of severe forms of COVID-19, they do not completely prevent infection, and their long-term effectiveness is limited, especially for newly emerging variants [4]. Another major challenge is the cost of producing vaccines to meet the demand in low-income countries [5]. It has been reported that most populations in some countries are reluctant to be vaccinated [6]. However, the presence of numerous CoVs suggests that zoonotic transmission will continue to occur [7], and new pandemic-potential viruses are emerging [8], creating major global public health concerns. Thus, investigating antivirals that aim to disrupt the viral entry process could aid in the development of therapeutic strategies for both pre- and post-exposure prophylactic treatments of COVID-19. Additionally, it may lead to the identification of potential anti-CoV drug candidates, preparing us for future pandemics.

The SARS-CoV-2 Spike (S) protein plays a crucial role in the viral entry into the host cells. The binding of the receptor-binding domain (RBD) of the S protein (S-RBD) to the cellular angiotensin-converting enzyme 2 (ACE2) receptor represents the first event (in both the endosomal and nonendosomal pathway) in the viral replication cycle and presents opportunities for prophylactic intervention [9]. There are already some vaccines, small peptides, convalescent serums, monoclonal antibodies, etc. [10,11], being developed or clinically applied to inhibit the binding of ACE2 and S-RBD; however, the immunogenicity of biologics, cost issues, and patient compliance limit their use, while small-molecule inhibitors that prevent the interaction of S-ACE2 are increasingly developed due to the advantages of the non-existence of such issues and being less affected by the mutation of the S protein [12].

And various strategies like virtual screens [13,14,15,16,17], ELISA [18,19], in-house immunofluorescence assays [20], bioluminescence and homogeneous SARS-CoV-2 RBD-ACE2 interaction assays [21], and pseudoviral-based systems [22] have now been adopted to identify potential entry inhibitors. But problems such as the requirement of a large amount of purified protein, the demand of further confirmation, high screening costs, long operation time, and the biosafety level (BSL) of the laboratory have limited their widespread application, respectively. In this research, we designed a quick and robust assay for measuring the S-RBD attachment to the ACE2 receptor by leveraging the Yeast-Two Hybrid system (Y2H) within a biosafety level 1 (BSL-1) laboratory setting to identify potential entry inhibitors against SARS-CoV-2 infection. Our assay not only overcomes the problems mentioned above but also allows the interaction of ACE2 and S-RBD to be studied in living yeast cells, which provides a more biologically relevant environment compared to in vitro methods. Using this high-throughput screening (HTS) assay, we screened the entry inhibitors from 3500 compounds and identified IMB-1C as a candidate by preventing the binding of S-RBD to ACE2 and blocking pseudotyped SARS-CoV-2 entry and infection.

## 2. Results

### 2.1. SARS-CoV-2 S-RBD Interacted with ACE2 in the Y2H Assay

The Y2H system adopted exploits the modular nature of the yeast Gal4 transcription factor. The bait and prey proteins are fused either to the Gal4 DNA-binding domain (BD) or to the Gal4 activation domain (AD). When the bait and prey proteins interact within the yeast nucleus, they bring together AD and BD, and subsequently trigger the expression of downstream reporter genes, which include nonselective and colorimetric reporter genes, such as the β-galactosidide (β-gal), and selective reporter genes in charge of adenine or histidine biosynthesis, which complement the chromosomal mutation in a metabolic pathway. The interaction of individual bait protein with the prey protein triggers the reporter gene activation, enabling the yeast to grow on selective plates [23].

**Figure 1:**
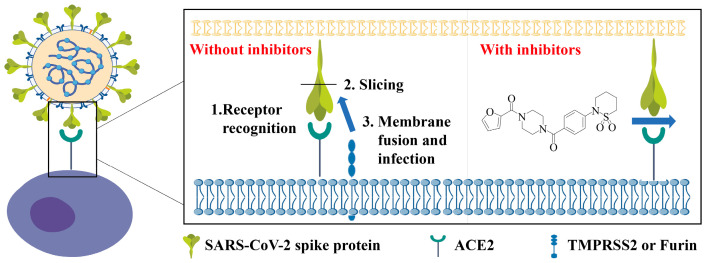
Illustrative representation of severe acute respiratory syndrome coronavirus 2 (SARS-CoV-2) binding to host cells through Spike (S)-angiotensin-converting enzyme 2 (ACE2) interaction. SARS-CoV-2 utilizes human ACE2 as an entry receptor to gain access into target cells. Small-molecule inhibitors that block S-mediated entry offer a promising blueprint for the development of therapeutic interventions.

**Figure 2:**
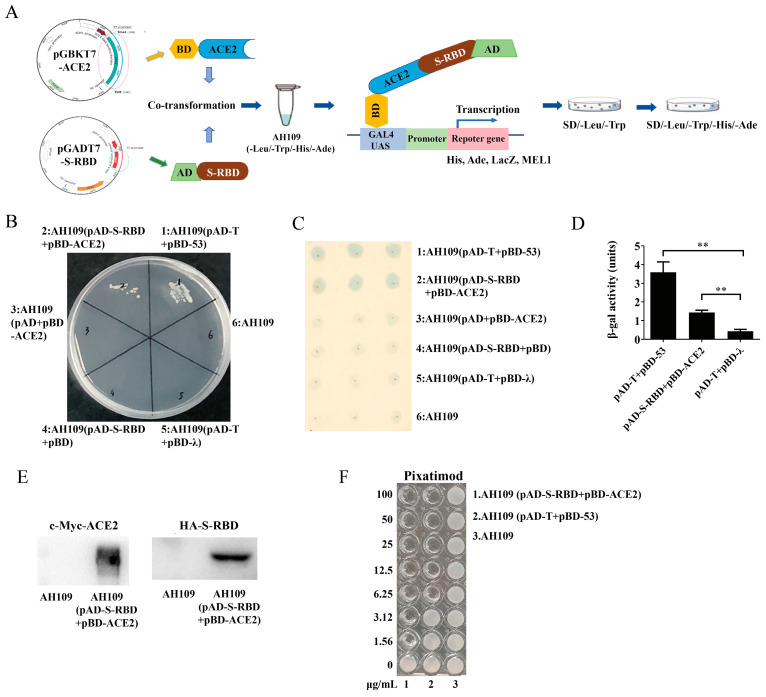
Establishment of the yeast-two hybrid (Y2H)-based high-throughput screening (HTS) model targeting S-RBD-ACE2 interaction. (**A**) Schematic diagram of the Y2H-based S-RBD-ACE2 binding assay. The interaction between ACE2 and S-RBD restores the function of the transcription factor Gal4, leading to the expression of the reporter genes *ADE2*, *HIS3*, and *LacZ*, which ultimately enables the recombinant Y2H system to grow in SD/-Leu/-Trp/-His/-Ade dropout medium. (**B**) The proliferation of AH109 and AH109 cells carrying various combinations of the Gal4 DNA-binding domain (BD) and the Gal4 activation domain (AD) fusion proteins (pAD-T+pBD-53, pAD-S-RBD+pBD-ACE2, pAD+pBD-ACE2, pAD-S-RBD+pBD, and pAD-T+pBD-λ) on an SD/-Leu/-Trp/-His/-Ade dropout plate. (**C**) Qualitative assessment of β-gal activity within the Y2H assay. Filter paper containing lysed colonies from selective plates were overlaid with X-gal buffer. Color change can be observed after overnight incubation. (**D**) Quantitative measurement of β-gal activity within the Y2H assay. Pelleted cells were resuspended in Z-buffer. After the addition of chloroform, SD, ONPG solution, and chilled Na_2_CO_3_ successively, absorbance was measured at a wavelength of 420 nm. Data were expressed as mean ± standard deviation of three independent experiments, each performed in triplicate. ** *p* < 0.01. (**E**) The expression of S-RBD and ACE2 in newly built Y2H assay detected by the expression of the corresponding vector-tagged proteins. (**F**) Evaluation of Y2H assay by Pixatimod. Inhibitory effect of Pixatimod on the interaction of S-RBD with ACE2 was tested within the Y2H assay.

SARS-CoV-2 entry into host cells serves as a crucial factor influencing viral infectivity and pathogenesis [24]. It begins with the recognition of host cell receptor ACE2 by viral S-RBD. Then, after the priming of the S protein by proteases, the virus fuses its membrane with the host cell membrane (Figure 1). If there exist small-molecule inhibitors that can block the interaction between S-RBD and ACE2, it can prevent the virus from entering the host cells as illustrated in Figure 1. Due to the immense biomedical significance of the S-RBD-ACE2 interaction for viral attachment, the interaction between the two proteins was first confirmed by the Y2H system. Given that RBD is the domain responsible for receptor recognition and binding to ACE2, SARS-CoV-2 S-RBD was fused to AD in pGADT7 plasmid, which led to the generation of pAD-S-RBD (Figure S1A) and the full-length human ACE2 was fused to BD in pGBKT7 plasmid resulting in the generation of pBD-ACE2 (Figure S1B). The physical direct interaction between S-RBD and ACE2 within yeast cells triggered the production of a functional Gal4 transcription factor, subsequently driving the expression of reporter genes (*HIS3*, *ADE2*, and *LacZ*) that are under the control of the GAL promoter (Figure 2A). As a result, the Y2H reporter strain AH109 harboring both pAD-S-RBD and pBD-ACE2 could grow in the synthetic yeast media (SD/-Leu/-Trp/-His/-Ade) lacking leucine/tryptophan/histidine/adenine. The same growth condition can be tested with the positive control, AH109 (pAD-T+pBD-p53), which contains an independent yeast-interacting protein pair, whereas the negative control, AH109 (pAD-T+pBD-λ), failed to grow in the dropout plate. Due to the possibility of self-activation, all six yeast strains (AH109 (pAD-T+pBD-53), AH109 (pAD-S-RBD+pBD-ACE2), AH109 (pAD+pBD-ACE2), AH109 (pAD-S-RBD+pBD), AH109 (pAD-T+pBD-λ), and AH109 were placed on solid SD/-Leu/-Trp/-His/-Ade media. Only the growth of AH109 (pAD-T+pBD-53) and AH109 (pAD-S-RBD+pBD-ACE2) could be detected, proving the existence of interaction between S-RBD and ACE2, along with T and 53, in the meantime excluding the possibility of self-activation (Figure 2B).

Apart from evaluating the growth capacity in dropout media due to the reconstitution of functional transcription factors and the simultaneous activation of the reporter gene, the interaction between S-RBD and ACE2 can also be confirmed through qualitative and quantitative measurements of β-galactosidide (β-gal) activity, encoded by the *Escherichia coli lacZ* gene, which was reconstituted by the interaction between two proteins [25]. In the colony filter lift assay with X-gal serving as the substrate (Figure 2C), the blue coloration caused by the catalytic activity of β-gal could only be observed by AH109 (pAD-S-RBD+pBD-ACE2) and AH109 (pAD-T+pBD-p53). Meanwhile, the utilization of O-nitrophenyl β-D-galactosidide (ONPG) cleavage in a liquid assay (Figure 2D) based on the catalytic activity of β-gal also indirectly proved the existence of interaction between S-RBD and ACE2. We also employed Western blot analysis to confirm the expression of S-RBD and ACE2 in yeast cells, as illustrated in Figure 2E.

Pixatimod has been shown to directly inhibit the S-RBD binding to ACE2 [26]. Therefore, we tested its inhibitory effects on the interaction of S-RBD with ACE2 in the Y2H assay. A dose–response was observed, with Pixatimod showing a minimum inhibitory concentration (MIC) of 1.56 μg/mL against AH109 (pAD-S-RBD+pBD-ACE2) and 6.25 μg/mL against AH109 (pAD-T+pBD-p53) (Figure 2F), which demonstrated the successful construction of the model for subsequent application to the HTS of S-ACE2 interaction inhibitors.

### 2.2. HTS for Entry Inhibitors from a Compound Library

**Figure 3:**
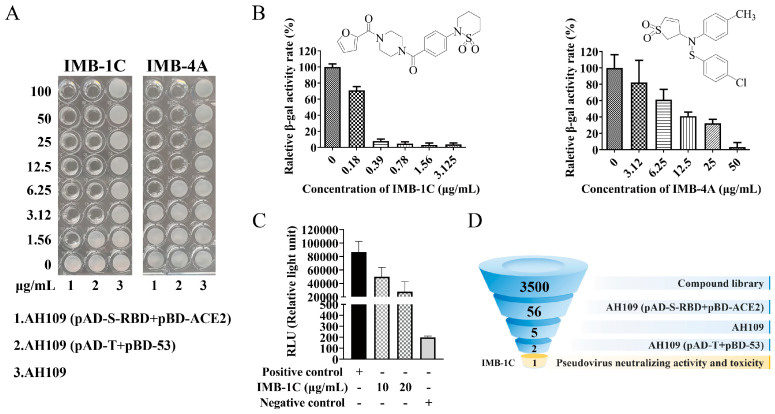
HTS of inhibitors targeting S-RBD-ACE2 interaction. (**A**) Growth inhibition within Y2H assay by S-RBD-ACE2 interaction inhibitors. AH109 cells with indicated plasmids (pAD-S-RBD+pBD-ACE2 and pAD-T+pBD-53) were treated with hit compounds IMB-1C and IMB-4A (from 0.78 to 100 µg/mL) in 96-well plates in SD/-Leu/-Trp/-His/-Ade dropout medium for 48 h and the growth of cells is shown. (**B**) The inhibition of β-galactosidide (β-gal) activity of IMB-1C and IMB-4A against AH109 (pAD-S-RBD+pBD-ACE2) cells and their structures. The data show the ratios of β-gal activity of cells treated with compounds over that of untreated cells. The results are mean ± standard deviation (SD) from triplicated assays. (**C**) The inhibitory effect on SARS-CoV-2 pseudovirus entry into host cells by IMB-1C. Pseudoviruses and compounds, at concentrations of 10 and 20 μg/mL, were incubated at 37 °C for a period of 1 h prior to addition into HEK-293T-hACE2 cells for an additional 18 h incubation. Pseudotyped virus entry into HEK-293T-hACE2 was determined by measuring relative luciferase units (RLU) in cell lysates. IMB-1C showed concentration-dependent inhibition, whereas the negative control showed no significant effect. The results are from triplicated assays. (**D**) Overview of the screening process for inhibitors of S-RBD and ACE2 interaction. During the initial screening process, 3,500 compounds were evaluated for their ability to inhibit the interaction between S-RBD and ACE2, resulting in the selection of 56 compounds that completely halted the growth of AH109 (pAD-S-RBD+pBD-ACE2) at a concentration of 50 μg/mL. After excluding the ones with antifungal activity, there were 5 compounds left. Among them, only 2 compounds exhibited more potent inhibitory effects on AH109 (pAD-S-RBD+pBD-ACE2) compared to AH109 (pAD-T+pBD-53) with MICs more than 2 times. Ultimately, IMB-1C was chosen as the most promising candidate due to its demonstrated antiviral activity exceeding 50% at a concentration of 40 μg/mL against pseudoviruses.

To identify the chemical compounds that can block the interaction between ACE2 and S-RBD, AH109 (pAD-S-RBD+pBD-ACE2) was utilized to perform a comprehensive screening of the compound library obtained from the National Center for Screening Novel Microbial Drugs, China. Among the 3500 compounds in the primary screening, 56 compounds showed growth-inhibiting effects against the AH109 (pAD-S-RBD+pBD-ACE2) strain at the concentration of 50 µg/mL. During the second round of screening, five compounds did not concurrently inhibit the growth of AH109, which was plated in parallel to serve as a control, enabling the detection and elimination of potentially toxic antifungal compounds. Further analyses for the selectivity of protein–protein interaction (PPI) inhibition were performed by comparing the MICs against AH109 (pAD-S-RBD+pBD-ACE2) and AH109 (pAD-T+pBD-53). Those with MICs for AH109 (pAD-T+pBD-53) more than twice that for AH109 (pAD-S-RBD+pBD-ACE2) were considered as specific inhibitors of the S-RBD-ACE2 interaction. After this round of screening, IMB-1C and IMB-4A were identified as the candidates (Table 1). A representative panel demonstrating different inhibition profiles toward the growth of AH109 (pAD-S-RBD+pBD-ACE2) and AH109 (pAD-T+pBD-53) by these two compounds is presented in Figure 3A. The MICs showed a 2-fold difference among AH109 (pAD-S-RBD+pBD-ACE2) and AH109 (pAD-T+pBD-53), with IMB-1C exhibiting a lower MIC for AH109 (pAD-S-RBD+pBD-ACE2), which was 1.56 µg/mL compared with IMB-4A, which was 6.25 µg/mL. They were further subjected to the β-gal quantitative experiment. Both treatments resulted in a notable decrease in β-gal activity in a dose-dependent manner (Figure 3B). IMB-1C and IMB-4A showed a clear inhibition of β-gal activity at 0.39 and 6.25 µg/mL, respectively (Figure 3B). These two compounds, reflecting a yield of approximately 0.056% from the starting library, were selected for subsequent experiments.

### 2.3. Inhibition of SARS-CoV-2 Pseudovirus Viral Entry

To test whether the inhibition of S-RBD-ACE2 interaction by IMB-1C and IMB-4A could block pseudovirus viral entry, HEK-293T-hACE2 and pseudoviral particles bearing the S protein of SARS-CoV-2 and a luciferase reporter gene were applied as the viral entry model system. It was observed that only IMB-1C reduced the entry of pseudo particles facilitated by the SARS-CoV-2 S protein at tested concentrations, yielding an inhibition rate of 68.10% at 20 µg/mL and 42.46% at 10 µg/mL, respectively (Figure 3C), but such inhibition could not be observed for the vehicle control and IMB-4A. These results showed that the inhibition of the S-RBD-ACE2 interaction in Y2H assay by IMB-1C was similarly effective in blocking the viral entry of SARS-CoV-2 pseudovirus. To eliminate the possibility that the observed inhibitory activity was due to the decreased cell viability induced by IMB-1C, the cell viability was measured via CCK-8 assay. The result showed that treatment with a concentration of up to 50 µg/mL for 48 h did not affect the cell proliferation of Vero, HEK-293T, and Huh-7 cells, suggesting that the entry inhibitory effect was not an artifact of the experimental system but was a direct result of the IMB-1C’s inhibition of pseudovirus infection in vitro (Table 2). Due to the lack of inhibitory activity against pseudovirus infection and cell cytotoxicity (survival rate only 18% for HEK-293T and 25% for Huh-7 at the concentration of 50 µg/mL), IMB-4A was excluded in subsequent experiments (Table 2). The whole screening process is depicted in Figure 3D.

### 2.4. IMB-1C Effectively Inhibited SARS-CoV-2 S-Mediated Cell–Cell Fusion

**Figure 4:**
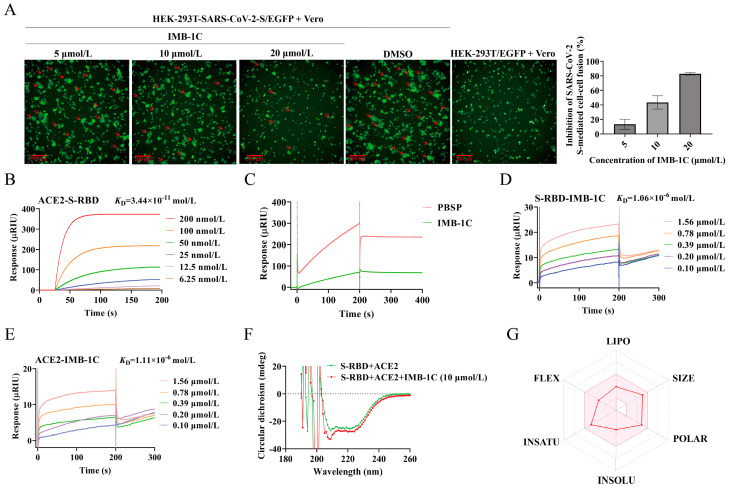
The inhibition profile of IMB-1C against the interaction between S-RBD-ACE2 of SARS-CoV-2. (**A**) IMB-1C inhibited SARS-CoV-2-S-mediated cell–cell fusion. Vero cells (target cells) were co-cultured with HEK-293T/EGFP/SARS-CoV-2-S (effector cells) in the absence or presence of IMB-1C (5, 10, and 20 μmol/L). After co-incubation at 37 °C for 12 h, syncytium formation was observed under a high-content analysis system and the inhibition rate was calculated. The red arrow represents the fused cells. (**B**) Detection of ACE2-S-RBD binding affinity by surface plasmon resonance (SPR). S-RBD was coated on a SAM chip and serially concentrated solutions of ACE2 (from 6.25 nmol/L to 200 nmol/L) were injected into the chamber. The change in response units is shown. (**C**) IMB-1C blocked the binding of ACE2 with S-RBD in vitro. S-RBD was fixed and sealed onto the SAM chip, and IMB-1C (0.40 μmol/L) flowed over the SAM chip. After ACE2 was injected, the change in response units is shown. PBSP was injected as the negative control. (**D**) SPR analysis indicated SARS-CoV-2 S-RBD was the binding partner of IMB-1C. Serially concentrated solutions of IMB-1C (0.10~1.56 µmol/L) were injected into the chamber with a SAM chip coated with S-RBD. The change in response units was shown. (**E**) SPR analysis indicated that ACE2 was also the binding partner of IMB-1C. Serially concentrated solutions of IMB-1C (0.10~1.56 µmol/L) were injected into the chamber with a SAM chip coated with ACE2. The change in response units is shown. (**F**) Conformational change in S-RBD+ACE2 complex assessed by Circular dichroism (CD) spectroscopy. The secondary structure of S-RBD+ACE2 complex with or without IMB-1C (10 µmol/L) in PBS was examined by CD spectroscopy. Double minima at 208 nm and 222 nm are revealed. (**G**) SwissADME analysis for IMB-1C. Radar plot depicts the ADME data of IMB-1C. The pink region signifies the desired range for each property (clockwise from top, lipophilicity: XLOGP3 between −0.7 and +5.0, size: MW between 150 and 500 g/mol, polarity: TPSA between 20 and 130 Å2, solubility: log S ≤ 6, saturation: fraction of carbons in the sp3 hybridization ≥ 0.25, and flexibility: ≤9 rotatable bonds).

To further confirm that the observed effect was due to the viral entry inhibition, IMB-1C was tested through the S-ACE2 attachment assay in live cells. This assay comprised two primary components, Vero cells that express ACE2 on its surface, serving as the target cells, and HEK-293T cells transfected with either EGFP (designated as HEK-293T/EGFP) or SARS-CoV-2-S/EGFP (designated as HEK-293T/SARS-CoV-2-S/EGFP) possessing ACE2-binding activity, which function as effector cells. The two kinds of effector cells were incubated with Vero, respectively, and changes in the fluorescence intensity were monitored. In contrast, the strong and concentrated fluorescence signal detected by the co-incubation of Vero with HEK-293T/EGFP was weakened and dispersed following the co-incubation of Vero with HEK-293T/SARS-CoV-2-S/EGFP, which was ascribed to the recognition of the ACE2 and the S protein during the fusion process. The addition of IMB-1C into the co-incubation of Vero with HEK-293T/SARS-CoV-2-S/EGFP restored the fluorescence activity, and the syncytium formation was somewhat blocked in a dose-dependent manner, suggesting its effectiveness in blocking the S protein attachment to ACE2 (Figure 4A).

### 2.5. IMB-1C Blocked S-RBD-ACE2 Interaction Through Binding with Both S-RBD and ACE2

The study of the possible mechanisms by which IMB-1C inhibited S-RBD-ACE2 interaction was conducted through surface plasmon resonance (SPR). The results revealed a strong binding affinity between S-RBD and ACE2 with the equilibrium dissociation constant (*K*_D_) in the low nanomolar range of 3.44 × 10^−11^ mol/L (Figure 4B). In the presence of 0.40 µmol/L of IMB-1C, the signal value decreased, indicating a blockage of ACE2-S-RBD interaction (Figure 4C). To elucidate the underlying mechanism, we evaluated the binding possibility of IMB-1C to S-RBD and ACE2. Figure 4D, E implied that IMB-1C could bind to both S-RBD and ACE2, with medium binding affinity for S-RBD (*K*_D_ = 1.06 × 10^−6^ mol/L) and ACE2 (*K*_D_ = 1.11 × 10^−6^ mol/L). These findings suggested that IMB-1C could block the ACE2-S-RBD interaction by directly binding to SARS-CoV-2 S-RBD and ACE2 at the binding interface.

### 2.6. Circular Dichroism (CD) Spectroscopy Assay

To further clarify the effect of IMB-1C on the inhibition of S-RBD+ACE2 interaction, an investigation into the impact of IMB-1C on the secondary structure of S-RBD+ACE2 complex was conducted by CD spectroscopy. As shown in Figure 4F, the percentage content of α-helix, β-fold, and β-turn structure was 6.27%, 51.53%, and 16.80%, respectively. For S-RBD+ACE2 treated with IMB-1C, the content of β-fold decreased by 9.46%, whereas the content of α-helix increased by 6.85%, which indicated that IMB-1C influenced the secondary structure of the S-RBD+ACE2 complex (Table 3).

### 2.7. Druggability Evaluation of IMB-1C

An exploration of the pharmacogenetic properties and drug likeness of compounds constitutes the essential aspects of drug development. Absorption, Distribution, Metabolism, Excretion and Toxicity (ADMET) prediction manifested that IMB-1C exhibited physicochemical properties in line with Lipinski’s rules and possessed good bioavailability, with a value of 0.55, due to its high solubility and efficient gastrointestinal absorption (Figure 4G). Since IMB-1C was unable to penetrate the blood–brain barrier, it will not exert any influence on the central nervous system. Nonetheless, it acted as an inhibitor for several drug-metabolizing enzymes, such as CYP3A4, CYP2C19, and CYP2C9, and was also a substrate for P-glycoprotein (P-gb), potentially leading to drug–drug interactions (Table 4). Therefore, there remains room for further structural modification.

## 3. Discussion

The recent COVID-19 pandemic has highlighted the necessity to bolster our preparedness for similar disease outbreaks. The most effective measure for preventing viral diseases is vaccination. However, vaccine development typically takes a long time and consumes significant financial and labor resources. It is also affected by several hurdles, including global implementation and distribution bottlenecks [27]. Therefore, safer and more accessible strategies need to be developed for combating disease outbreaks in the future.

Any stage in the life cycle of a virus holds the potential to be a drug target [28]. Among them, viral attachment to the host cells is the initial step and represents a compelling point of intervention, with high prospective to prevent or treat viral infections [29]. And the formation of the S-ACE2 complex is the primary contact point for the entry of SARS-CoV-2 into host cells [10]. Moreover, SARS-CoV-2 and other CoVs have similar infection mechanisms [30]. This applies particularly to SARS-CoV and CoV-NL63, as they both utilize the same human ACE2 receptor to gain entry into host cells. This strategy was first demonstrated by Hsiang et al. In their study, they announced the successful inhibition of interaction between SARS-CoV S and ACE2 using small peptides, as demonstrated through an ELISA assay [10]. In addition to peptides, vaccines, monoclonal antibodies, and convalescent sera from survivors, small-molecule inhibitors can also block the viral-host interaction [18,31,32]. However, the appropriate titer of the convalescent-phase sera antibody required to achieve clinically significant therapeutic efficacy in inhibiting viral infection remains to be determined. Research into MERS-CoV indicated that the sera collected from patients who had recovered from the infection lacked adequate antibody titers for therapeutic purposes [11]. Antibodies and peptides, like all protein therapies, face challenges such as limited solubility, unsuitability for oral or inhaled routes of administration, and immunogenicity, in addition to their highly specific targeting nature [18]. As foreign proteins, they have the potential to act as antigens and trigger robust immune responses in certain patients [33,34,35], a problem that is further compounded by their prolonged elimination half-lives [36]. Even among the therapeutics approved by FDA, more post-market safety issues have been reported for biologics compared to small-molecule drugs [37]. Moreover, compared with biologics, small molecules are more economically sustainable, more accessible to patients, more stable as they rarely require specialized storage conditions, and their behavior in the human body is usually predictable and easy to be administered [38]. Therefore, small molecules may provide alternative solutions with superior performance.

Currently, virtual screening [13,14,15,16,17], molecular docking [39,40], ELISA [18,19], in-house immunofluorescent assay [20], bioluminescent and homogeneous SARS-CoV-2 RBD and ACE2 interaction assays [21], as well as pseudovirus-based systems have been employed to identify prospective S-ACE2 inhibitors [22]. Virtual screening relies on the already known molecules, saving time and money. However, this approach requires the integration of several databases to create a library of chemicals. Such a library is queried based on criteria like the resemblance in electrostatic properties and overlapping chemical structures [41,42]. Molecular docking involves simulating the binding between potential drug candidates with the biological targets (enzymes or proteins), with the aim of finding a conformation that provides a good interaction [43,44,45,46]. However, simulation differs from the prototype and lacks practicality [19]. Moreover, the activity of the predicted binding needs to be verified experimentally. Pseudoviruses are not easily accessible and require special handling in BSL-2 facilities [47]. ELISA and ELISA-based assays are relatively expensive, cumbersome, and require large amounts of purified protein. These factors limit and delay the development of efficient anti-viral candidates. Considering the possibility of the emergence of new virus strains or another pandemic, drugs that offer a rapid and adequate response need to be developed. The Y2H-based assay, which has several advantages, including minimal hands-on time, being rapid, no requirement for a BSL-1 laboratory facility, the use of standard laboratory equipment, and reliance on cheap reagents, has been utilized in HTS to facilitate drug discovery, enabling the identification of PPI inhibitors within a eukaryotic cellular context [48]. However, one significant barrier to the use of Y2H for drug HTS is that yeast cells are relatively impermeable to a broad spectrum of macromolecules. Therefore, physical and chemical genetic techniques should be used to enhance the permeability of yeast membranes.

## 4. Materials and Methods

### 4.1. Cell Culture

African green monkey kidney cells (Vero), Human Hepatocellular Carcinoma Cells (Huh-7), and Human Embryonic Kidney 293T (HEK-293T) cells were maintained in our laboratory. Cells were cultured in Dulbecco’s Modified Eagle’s Medium (DMEM, Hyclone, Logan, UT, USA) containing 10% fetal bovine serum (FBS, Gibco, Waltham, MA, USA) at 37 °C under 5% CO_2_ in a humidified environment.

### 4.2. Construction of Y2H Assay

The Y2H assay was constructed following a previously reported method [49]. Briefly, DNA fragments encoding S-RBD (aa319-541) [50] was cloned into the prey vector pGADT7 (AD, Clontech, Mountain View, CA, USA) to construct a recombinant plasmid pAD-S-RBD, while the full-length of ACE2 were cloned into the bait vector pGBKT (BD, Clontech, Mountain View, CA, USA) to produce a plasmid pBD-ACE2, both using *Sma*I and *Ps*tI restriction sites. The bait vector contained a leucine synthesis gene for selecting yeast cells. Meanwhile, the prey vectors contained a tryptophan synthesis gene for selecting yeast cells. The successful transformation of yeast cells with both bait and prey constructs (pAD-S-RBD+pBD-ACE2) was verified using the plating method and growing yeast cells on synthetically defined (SD) agar plates (Clontech, Mountain View, CA, USA) without leucine and tryptophan (SD/-Leu/-Trp) (transformation control), and SD plates lacking leucine, tryptophan, adenine, and histidine (SD/-Leu/-Trp/-Ade/-His) (interaction control).

### 4.3. β-Gal Colony Filter Lift Assay

The expression level of β-gal was qualitatively determined using the β-gal colony filter lift assay. In brief, cell colonies were obtained from selective plates, immersed in liquid nitrogen and thawed at 30 °C repeatedly, until they were completely lysed. The lysate was transferred onto the filter paper overlaid with 5-bromo-4-chloro-3-in-dolyl-β-d-galactoside (X-gal) buffer (100 mmol/L Na_2_HPO_4_, 35 mmol/L NaH_2_PO_4_, 10 mmol/L KCl, and 1 mmol/L MgSO_4_, pH 7.0, 0.4 mg/mL X-gal and 0.27% β-mercaptoethanol). The filters were incubated overnight at room temperature (RT) to observe the color change.

### 4.4. ONPG Assay

The ONPG assay was conducted following the protocol of the β-gal activity quantitative detection kit (GENMED SCIENTIFICS INC. USA, Arlington, MA, USA). The principle is based on the chemical reaction that the colorless substrate ONPG can be hydrolyzed by β-gal to generate o-nitrophenol (ONP) with a bright-yellow appearance. Briefly, cells were pelleted in Eppendorf^®^ tubes and resuspended in Z-buffer. Chloroform and 0.2% SDS were added to the cells in the tubes and mixed by briefly vortexing, followed by incubation at 28 °C for 5 min. Next, the ONPG solution was added for further incubation. The reaction was terminated after 10 min for p53-T and after 25 min for all other samples by the addition of chilled Na_2_CO_3_. Absorbance was recorded at 420 nm. Results were normalized against the cell density and incubation time using the following formula:

$$U=\frac{{OD420}_{sample}-{OD420}_{blank}}{OD600\times t\times V}$$

Here, ‘t’ represents the incubation time duration, measured in min, while ‘V’ denotes the volume of the cell cultures utilized for the assay, expressed in mL. The experiments were repeated three times.

### 4.5. Western Blot Analysis

The expression level of ACE2 and S-RBD proteins was verified by Western blot. Briefly, yeast cells were centrifuged and resuspended in buffer (50 mmol/L Tris, 150 mmol/L NaCl, 1% Nonidet P-40, 0.5% sodium deoxycholate, 0.1% SDS, pH 7.5) and lysed with high-pressure cell cracker. The resulting supernatants were separated on polyacrylamide gels and transferred onto a polyvinylidene difluoride (PVDF, Millipore, Burlington, MA, USA) membrane. The membrane was blocked and incubated with anti-Myc/HA and HRP-labeled IgG to detect S-RBD and ACE2 expression in a newly built Y2H system. The signal was developed using Immobilon Western chemiluminescent HRP substrate (Millipore, Burlington, MA, USA).

### 4.6. HTS Assay

The Chemdiv screening compound library used for HTS assay in this research is a combination of synthetic compounds from Enamine (Kyiv, Ukraine) and Life Chemicals Inc. (Burlington, ON, Canada), which can be purchased from Shanghai Topscience Biotechnology Co., Ltd., China. The compounds were stocked at 10 mg/mL in DMSO and used for the screening assays as described [51]. In summary, in the primary screening, freshly cultured AH109 (pAD-S-RBD+pBD-ACE2) cells (OD_600_ = 0.8) were diluted 1000-fold in SD/-Leu/-Trp/-Ade/-His in 96-well plates. Next, 199 μL of the diluted culture and 1 μL of tested compounds (the final concentration was 50 μg/mL) or DMSO were added into each well. The growth inhibition of the yeast cells was subsequently examined after 48 h at 30 °C, and compounds that could completely inhibit the growth of AH109 (pAD-S-RBD+pBD-ACE2) cells were selected as primary positive compounds. Compounds that completely inhibited the AH109 cells were considered to possess antifungal activity and were excluded. MICs of the left positive compounds against AH109 (pAD-S-RBD+pBD-ACE2) and AH109 (pAD-T+pBD-53) were further detected. The final concentrations of the compounds ranged from 100 μg/mL to 0.78 μg/mL with 2-fold dilutions between concentrations. The MIC endpoints were defined as the lowest concentration of compounds that could completely inhibit the growth of yeast. Compounds with MICs against AH109 (pAD-S-RBD+pBD-ACE2) lower than that of AH109 (pAD-T+pBD-53) by 2-fold were selected for further research.

### 4.7. Pseudovirus Entry Assay

HEK-293T-hACE2 cells were cultured in 96-well plates at the density of 3 × 10^4^ cells/well. Pseudoviruses and compounds (10, 20 μg/mL) were cultured at 37 °C for 1 h before they were added to the HEK-293T-hACE2 cells for further incubation for 18 h. The luminescence signal was recorded immediately using the luminescent microplate reader (BioTek Synergy HTX, Agilent Technologies, Santa Clara, CA, USA). For the purpose of determining the percentage of viral entry, luminescent data corresponding to the time point with the highest signal intensity in the negative control sample were chosen for downstream calculations. The percentage of neutralization (%) was calculated as follows:

$$x=(1-\frac{{luminescence\;signal}_{sample}}{{luminescence\;signal}_{negative\;control}})\times 100\%$$

### 4.8. In Vitro Cytotoxicity Test

The cytotoxicity of compounds against Vero, Huh-7, and HEK-293T cells was detected using the Cell Counting Kit-8. Briefly, the target cells were cultured at a density of 8000 cells per well in a 96-well plate containing DMEM enriched with 10% FBS. The cells were allowed to attach overnight, and the medium was replaced with a fresh serum-free DMEM containing 50 µg/mL of compounds. The cell viability was measured at 48 h post-drug treatment by the addition of the CCK-8 reagent (Biosharp, Hefei, Anhui, China), followed by incubation for 4 h. Finally, the absorbance of the cell culture was measured at 450 nm using a microplate reader (PerkinElmer, Waltham, MA, USA). The background absorbance of the blank media was also measured and subtracted from the sample reading. This experiment was repeated 3 times for each group. The obtained results were normalized to that of the control sample, which represented a cell viability of 100%.

### 4.9. Assessment of S Protein-Mediated Inhibition of Cell–Cell Fusion

The inhibitory activity of IMB-1C against the SARS-CoV-2 S-mediated cell–cell fusion was evaluated following a previous method [52]. Briefly, HEK-293T cells transfected with pAAV-SARS-CoV-2-S-IRES-EGFP plasmid encoding both the SARS-CoV-2 S protein and EGFP (effector cells) were co-cultured with Vero cells expressing the human ACE2 receptors on the membrane surface (target cells) in the absence or presence of IMB-1C at the final concentrations of 5, 10, and 20 μmol/L at 37 °C for 12 h, respectively. The formation of syncytium was observed under high-content analysis systems (PerkinElmer, Waltham, MA, USA) and the inhibition rate was calculated.

### 4.10. SPR Analysis

#### 4.10.1. Detection of ACE2-S-RBD Interaction

The SPR assay was performed to evaluate the ACE2-S-RBD interaction using the SAM chip (Reichert, Depew, NY, USA) by the Biacore™ S200 system (GE, Boston, MA, USA) in a PBSP running buffer (0.2 mol/L Phosphate buffer, 27 mmol/L KCl, 1.37 mol/L NaCl, 0.5% Surfactant P20). S-RBD was fixed onto the activated SAM chip at a rate of 5 μL/min. This was followed by a dilution of ACE2 to prepare different concentrations (6.25~200 nmol/L). The solution was allowed to flow through the surface of the chip at a flow rate of 30 μL/min. The results were analyzed using Biacore S200 Evaluation Software 1.1.1 (GE Healthcare, Chicago, IL, USA).

#### 4.10.2. Blockage of ACE2-S-RBD Interaction by IMB-1C

Once the S-RBD was fixed and sealed onto the SAM chip, IMB-1C (0.40 μmol/L) was allowed to flow through the SAM chip. Then, ACE2 was injected. For the negative control, the PBSP buffer was injected instead of IMB-1C, followed by the injection of an equal amount of ACE2.

#### 4.10.3. Measurement for the Binding Affinity Between IMB-1C and ACE2 or S-RBD

The binding affinity between IMB-1C and S-RBD or ACE2 was measured by immobilizing either S-RBD or ACE2 onto the surface of a SAM biosensor. IMB-1C was then added to the running buffer (PBSP). The obtained sensogram response data were analyzed using the Biacore S200 Evaluation Software 1.1.1 (GE Healthcare, Chicago, IL, USA), and the *K*_D_ was calculated. Each experiment was performed at least 3 times.

### 4.11. CD Spectroscopy Assay

S-RBD and ACE2 were mixed in PBS to achieve a final concentration of 1.47 μmol/L. Then, the mixture was treated with either IMB-1C at a concentration of 10 μmol/L or DMSO for a duration of 30 min at RT. CD spectral scanning in the ultraviolet region (190–260 nm) was performed with a CD spectrometer. Samples were scanned 3 times with a time constant of 1 s and a bandwidth of 1 nm. The CDNN software 2.1 was utilized to process and deconvolute the CD data, enabling the determination of the relative quantities of α-helix, β-turn, β-sheet, and random coil structures.

### 4.12. SwissADMET Prediction for IMB-1C

The SwissADME server serves as an online resource for predicting pharmacologically important characteristics and physically pertinent properties of chemical compounds. Initially, ChemDraw Ultra 14.0 (CambridgeSoft, Cambridge, MA, USA) was utilized to transform the structures of the hit compounds into the SMILES format. Subsequently, the “run to” process was initiated to predict the pharmacokinetics of IMB-1C.

### 4.13. Statistical Analysis

Statistical analysis was performed using GraphPad Prism 9.0 (GraphPad Software, San Diego, CA, USA). All the experiments were performed in triplicate, and the results were expressed as mean ± standard deviation (SD). Data between groups were compared with the two-tailed unpaired Student’s *t*-test. *p* < 0.05 was considered significant and expressed as *, *p* < 0.01 as **, and *p* < 0.001 as ***.

## 5. Conclusions

In this study, we used the Y2H system to establish an experimental assay for fast and cost-efficient HTS for S-ACE2 attachment inhibitors. The identified small molecule IMB-1C showed good potential to inhibit the S-RBD-ACE2 interaction, preventing pseudotyped SARS-CoV-2 entry into the host. Further in vitro and in vivo experiments are needed to explore its potential clinical use. In summary, our assay may serve as a backup assay, which is expected to promote the development of anti-viral inhibitors.

## 6. Patents

There is a patent resulting from the work reported in this manuscript.

## Figures and Tables

**Table 1:** Minimum Inhibitory Concentrations (MICs) (μg/mL) of IMB-1C and IMB-4A in Y2H assay.

MIC (μg/mL)	IMB-1C	IMB-4A
AH109 (pAD-S-RBD+pBD-ACE2)	1.56	6.25
AH109 (pAD-T+pBD-53)	3.125	12.5

**Table 2:** Cell survival rates of IMB-1C and IMB-4A at 50 μg/mL.

Survival Rate (%)	Vero	HEK-293T	Huh-7
IMB-1C	105	88	122
IMB-4A	71	18	25

**Table 3:** Circular Dichroism (CD) spectroscopy analysis for protein secondary structure content.

Sample	Secondary Structure Content (%)			
	α-Helix	β-Fold	β-Turn	Random
S-RBD+ACE2	6.27	51.53	16.80	29.53
S-RBD+ACE2+IMB-1C	13.12	42.07	15.97	32.93

**Table 4:** Absorption, Distribution, Metabolism, Excretion and Toxicity (ADMET) profile of IMB-1C.

Lipinski’s Rules	GI Absorption	Bioavailability	BBB Permeant	P-gb Substrate	CYP Enzymes’ Inhibitors
Yes	High	0.55	No	Yes	CYP2D6 (−), CYP3A4 (−), CYP2C19 (+), CYP2C9 (+), CYP3A4 (+)

## Data Availability

All data are available within the manuscript.

## Supplementary Materials

The supporting information can be downloaded at: https://www.mdpi.com/article/10.3390/ijms26020678/s1.
