# Supplementary material for: Establishment of a Yeast Two-Hybrid-Based High-Throughput Screening Model for Selection of SARS-CoV-2 Spike-ACE2 Interaction Inhibitors
Source: Int J Mol Sci. 2025 Jan 15;26(2):678. doi: 10.3390/ijms26020678 (PMC11765512; doi:10.3390/ijms26020678)
Supplement: Supplementary file 1 [file ijms-26-00678-s001.zip › ijms-3391923-supplementary.pdf]

Establishment of a Yeast Two-Hybrid-Based High-Throughput Screening Model for Selection of SARS-CoV-2 Spike-ACE2 Interaction Inhibitors

Dongsheng Li [1, 2, †], Baoqing You [1, †], Keyu Guo [1], Wenwen Zhou [1], Yan Li [1], Chenyin Wang [1], Xiaofang Chen [1], Zhen Wang [1], Jing Zhang [1, *], Shuyi Si [1, *]

[1]State Key Laboratory of Bioactive Substances and Functions of Natural Medicines, Institute of Medicinal Biotechnology, Chinese Academy of Medical Sciences & Peking Union Medical College, Beijing 100050, China;

[2] Research Team of Molecular Medicine, The First Clinical Medical School of Shanxi Medical University, Taiyuan 030001, China

† Dongsheng Li and Baoqing You contributed equally to this work.

* Correspondence: jingjingz@imb.pumc.edu.cn (J.Z.); sisy@imb.pumc.edu.cn (S.S.)

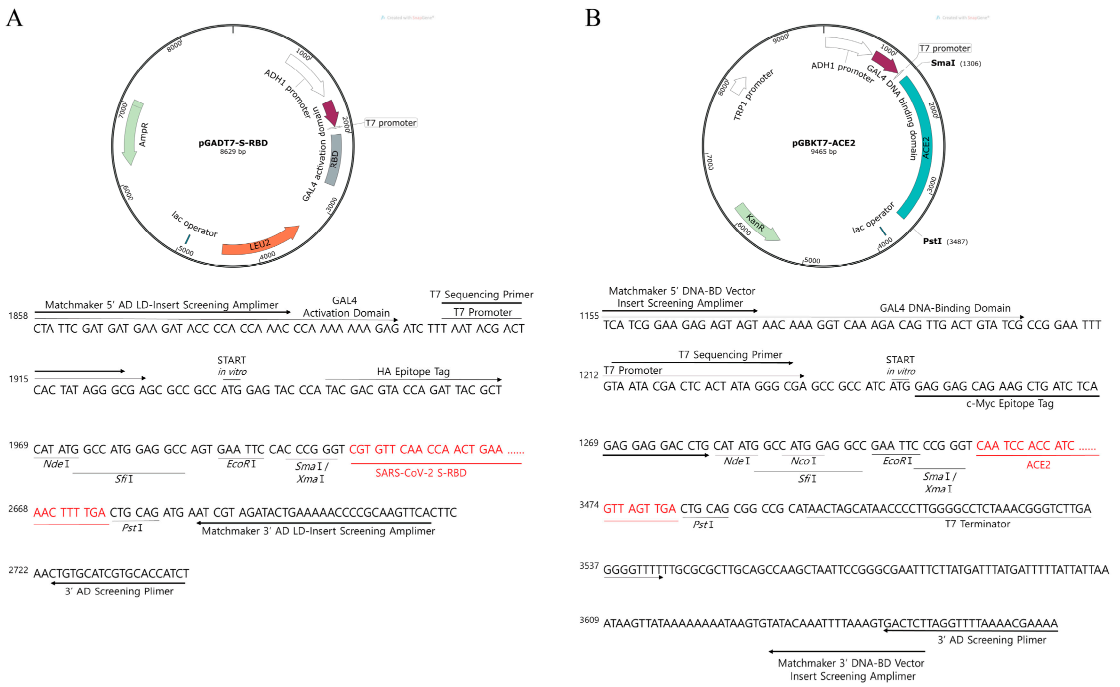

Figure S1. Construction of Yeast Two-Hybrid Recombinant Vector. (A) Vector map of recombinant pGADT7-S-RBD (pAD-S-RBD). S-RBD (aa319-541) was cloned into the prey vector pGADT7 via *Sma*I and *Pst*I. (B) Vector map of recombinant pGBKT7-ACE2 (pBD-ACE2). Full-length of ACE2 was cloned into the bait vector pGBKT7 via *Sma*I and *Pst*I.
